# Sucrose Isomerase Mutants’ Expression in *Bacillus subtilis* for Isomaltulose Production

**DOI:** 10.3390/microorganisms14040817

**Published:** 2026-04-02

**Authors:** Xiaoyang Liu, Dingfeng Chen, Yuhang Luo, Huirong Lv, Qian Wang, Zhongcang Qian, Zhengshun Wen

**Affiliations:** 1 School of Food and Pharmacy, Zhejiang Ocean University, Zhoushan 316022, China; liuxiaoyang@zjou.edu.cn (X.L.); chendingfeng2021@163.com (D.C.); luoyuhangtr@163.com (Y.L.); lvhuirong1215@163.com (H.L.); 2 Xianghu Laboratory, Hangzhou 311231, China; 3 College of Animal Sciences, Zhejiang University, Hangzhou 310058, China; emirate14@zju.edu.cn; 4 Institute of Ecological and Environmental Sciences, Taizhou Academy of Agricultural Sciences, Taizhou 318099, China; xiaobudian9116@126.com

**Keywords:** isomaltulose, sucrose isomerase, *Bacillus subtilis*, surface displaying, mutation

## Abstract

Isomaltulose is produced via sucrose isomerase catalysis by *Serratia plymuthica* A30. The enzyme was expressed in *Bacillus subtilis* using surface display, in combination with *Bacillus subtilis* spore coat CotC. The promoter was further selected and optimized to determine P_amyE_ as the most suitable promoter, while the spore coat protein assay reveals CotC as optimal. By scanning and analyzing the catalyst motifs with single-point mutation construction, a maximum isomaltulose yield of 27.21 mg/mL was recorded in the F181I-mutant enzyme. Another strain encoding the H363P-mutant reached a maximum yield of 20.84 mg/mL, while the k_cat_ value also increased from 17.64 to 24.80. Structural analysis showed that the F181I-mutant had higher thermostability, whereas the H363P-mutant had increased k_cat_. Both mutants displayed a 5-fold increase in isomaltulose yield with relatively simple construction procedures, making them suitable for high-level isomaltulose production.

## 1. Introduction

Sweeteners are commonly used in the food and drug industries to improve and enhance taste or to mask irritant and unpleasant odors [1]. Sucrose is traditionally and widely used as a natural sweetener because of its organoleptic properties; however, it has been associated with high calories and a high potential for diabetes and obesity, as well as cardiovascular diseases, according to multiple studies and trials, since the awareness of diet healthiness has arisen in recent years [2,3]. Fructose was primarily considered a sucrose alternative; however, several studies have shown that it has potential effects on diabetes and obesity similar to sucrose, as well as cytotoxic and genotoxic effects [4,5]. Consequently, a series of sucrose and fructose alternatives have been tested to identify the optimal candidate that performs well in terms of sweetness and non-diabetic properties. Among these alternatives, isomaltulose (D-glucopyranosyl-1, 6-fructose, or palatinose) has attracted significant attention because of its moderate sweetness and benefits in regulating blood sugar and intestinal health. Isomaltulose, which exists in several types of bee honey, has an appearance and taste similar to sucrose and a sweetening power equivalent to lactose and glucose. Since isomaltulose was approved for use as a sweetener by the FDA in 2017, its benefits and applications have been studied in various sweet foods and nutritional supplements [6,7]. Isomaltulose has low glycemic and insulinemic properties, showing a significantly lower effect on blood glucose and insulin release levels [8,9]. Recent research has also shown that isomaltulose has a moderate prebiotic effect by increasing the levels of beneficial microbiota, including *Faecalibacterium* and *Phascolarctobacterium*, and decreasing the levels of pathogens in the gut microbiota of rats [10].

**Figure 1:**
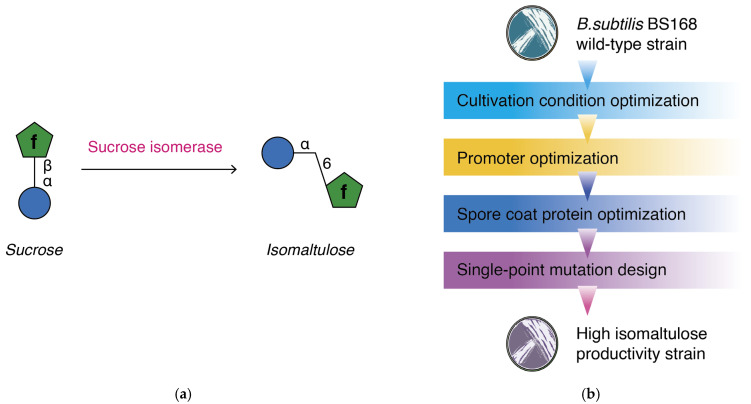
Production mechanism of isomaltulose and process of the current study. (**a**) Reaction mechanism of isomaltulose production from sucrose; (**b**) sketch map process of this study.

Among the various isomaltulose production techniques, microbial fermentation is commonly used because of its moderate conditions and high yield. The enzyme involved in the production of isomaltulose is sucrose isomerase (EC: 5.4.99.11) [11], which converts the glycosyl bond of sucrose from α-1, 2- to α-1, 6- (Figure 1a), and is expressed in a small number of microorganisms: *Protaminobacter rubrum* [12], *Erwinia rhapontici* [13], *Klebsiella planticola* [14], and *Serratia plymuthica* [15]. Among these bacteria, *Serratia plymuthica* exhibits a significantly higher initial activity and sucrose conversion rate of 75–80% [11]. In contrast to the high production of isomaltulose, the relatively rare abundance and specific cultivation conditions limit the broad application of *Serratia plymuthica* in isomaltulose production. Attempts have been made to heterologously express sucrose isomerase genes in engineered bacteria to reduce costs; however, wild-type enzymes are always involved in targeted mutations to adapt to a heterologous cytosolic environment and improve their catalytic activities. It has been reported that by constructing and importing plasmids encoding specific sucrose isomerase mutants into *Escherichia coli*, up to 80% of sucrose can be converted to isomaltulose within 24 h. The results showed that the conversion rate of sucrose isomerase expressed in *Escherichia coli* was equivalent to that of *Serratia plymuthica* as the host microbe [16]. Sucrose isomerase has also been modified and expressed in various yeast cells, exhibiting a maximum sucrose conversion rate of 73–85% [17].

*Bacillus subtilis* is considered to be an effective host for biochemical reactions. It is generally recognized as a safe species with limited hazards for food and drug production [18]. Owing to its environmental capability, *Bacillus subtilis* can endure extreme fermentation conditions, such as high pH, high temperature, and hazardous solvents. With its large library of expression vectors and simple initial metabolic pathways, it is relatively simple to construct de novo biosynthesis pathways and genetic optimizations in *Bacillus subtilis* without interference [19,20]. Unlike other Gram-positive bacteria, *Bacillus subtilis* lacks an outer membrane, allowing the location and arrangement of specific proteins at extracellular positions [21]. Surface display technology has progressed to ensure that proteins or enzymes are expressed on the cell membrane in a specific sequence [22,23]. Spore coat proteins, such as CotB, CotG, CotC, and CgeA, have been recombined with target proteins to construct a surface display system [24,25]. A series of bioactive compounds have been obtained from recombinant *Bacillus subtilis* strains, including recombinant proteins [26,27,28,29], antibodies [30,31], carbohydrates [32,33,34,35], terpenoids [36,37], and other compounds. Although sucrose isomerase has been constructed with certain surface display systems on yeast cells, particularly *Yarrowia lipolytica*, and has exhibited high conversion rates of up to 85–90%, its complex metabolic pathways often require gene editing of the genome in cases where side reactions may occur. The preparation of a surface display system on yeast cells requires stricter cultivation conditions than those for *Bacillus subtilis*, thereby limiting its applications [38,39]. This study focuses on the construction of a surface display of sucrose isomerase from *Serratia plymuthica* on *Bacillus subtilis*, while processing promoter and spore coat protein optimization, as well as enzyme single-point mutations and substrate concentration optimization, to obtain a high isomaltulose-producing *Bacillus subtilis* strain (Figure 1b).

## 2. Materials and Methods

### 2.1. Bacterial Strains and Media

*Bacillus subtilis* BS168 was used as the host for sucrose isomerase expression. LB medium consisted of 10 g/L tryptone, 5 g/L yeast extract, and 10 g/L NaCl. The growth medium consisted of 6 g/L tryptone, 12 g/L yeast extract, 12.5 g/L K_2_HPO_4_∙3H_2_O, and 2.5 g/L KH_2_PO_4_. A 10 mL trace element buffer solution was also added to the growth medium, which consisted of 4 g/L FeSO_4_∙7H_2_O, 4 g/L CaCl_2_, 1 g/L MnSO_4_∙H_2_O, 0.2 g/L NaMoO_4_∙2H_2_O, 0.2 g/L ZnSO_4_∙7H_2_O, 0.1 g/L AlCl_3_∙6H_2_O, 0.1 g/L CuCl_2_∙2H_2_O, and 0.05 g/L H_3_BO_3_ [40]. All reagents were purchased from Shanghai Macklin Biochemical Technology Co., Ltd. (Shanghai, China).

### 2.2. Sucrose Isomerase Site-Specific Mutants’ Construction

The complete nucleotide and protein sequences of the *Serratia plymuthica* A30 sucrose isomerase gene *smuA* were obtained from GenBank (AMSV01000038.1). The 3-dimensional molecular model of sucrose isomerase was built using Swiss-Model (https://swissmodel.expasy.org/interactive, last accessed on 16 March 2025). The mutant sites and models were analyzed and visualized using PyMOL v2.7. The molecular model of sucrose was obtained from PubChem (https://pubchem.ncbi.nlm.nih.gov/, last revised on 16 March 2025). The models were then imported into AutoDock 4 for catalyst center conformation and molecular docking analyses using genetic algorithms. The binding energies of the enzyme–substrate complexes were analyzed using AutoDock 4, whereas the protein–ligand interactions were analyzed using the Protein-Ligand Interaction Profiler (https://plip-tool.biotec.tu-dresden.de/plip-web/plip/index, last accessed on 20 March 2025) and visualized using PyMOL v2.7.

### 2.3. Plasmid Construction and Expression

The plasmid pMA09S1, containing the *Bacillus subtilis* spore coat protein gene *cotC* followed by the *Serratia plymuthica* A30 sucrose isomerase gene *smuA*, linked with a sequence encoding the GGGGS linker peptide, with the promoter P_hpaII_ and kanamycin resistance tag, was constructed based on the pMA09-H *Bacillus subtilis* plasmid (GenScript Corporation, Nanjing, China). Single-promoter plasmids were constructed using the ClonExpress Ultra One Step Cloning Kit (Vazyme, Nanjing, China). All single-promoter plasmids were based on the plasmid pMA09S1. Plasmid fragment A, which contained the complete sequence of pMA09S1 except for the promoter, was obtained by amplifying pMA09S1 using the primer pair P01/P02. Using the corresponding genomic DNA as templates, fragments of the promoters P_amyE_, P_sodA_, P_xylA_, P_lacI,_ and P_hag_ were obtained using the primer pairs P03/P04, P05/P06, P07/P08, P09/P10, and P11/P12, respectively. The promoter fragments were correctly joined to plasmid fragment A using the ClonExpress Ultra One Step Cloning Kit (Vazyme, Nanjing, China) to obtain plasmids pMA09S2, pMA09S3, pMA09S4, pMA09S5, and pMA09S6.

Optimal surface display protein plasmids were constructed using pMA09S2. Optimal surface display protein plasmids were constructed using pMA09S2, according to the workflow described above. Plasmid fragment B, comprising the complete sequence of pMA09S2 except *cotC*, was amplified using the primer pairs P13/P14. Five spore coat protein genes, *cotB*, *cotZ*, *oxdD*, and *cgeA*, were amplified using the genomic DNA of *Bacillus subtilis* and the primer pairs P15/P16, P17/P18, P19/P20, and P21/P22, respectively. The spore coat protein genes were correctly joined to plasmid fragment B using the same method to obtain plasmids pMA09S7, pMA09S8, pMA09S9, and pMA09S10. The sequences of the corresponding spore coat proteins and smuA were linked by a sequence encoding the GGGGS linker.

The mutant plasmids were constructed based on pMA09S2. The mutant plasmids were constructed using a similar technique as the one described above, while mutant primers were obtained using the Vazyme single-point mutation design tool (https://tool.vazyme.com:18002/cetool/singlepoint.html, last accessed on 11 July 2024). Each of the 19 designed smuA mutant sequences was used as a template for primer construction, obtaining primer pairs from PM1/PM2 to PM37/PM38. Each primer pair was used to amplify the plasmid pMA09S2 to obtain the corresponding mutant-encoding plasmids. All primers were constructed by Vazyme (Nanjing, China), and all plasmids were sequenced for verification using GenScript. The amplification targets of the primer pairs are listed in Table S1, and the sequences of each primer are listed in Table S2 in the Supplementary Materials.

*Bacillus subtilis* BS168 was transformed with each plasmid via electroporation to obtain the corresponding recombinant strains. Each recombinant strain was inoculated onto solid LB plates supplemented with kanamycin at a concentration of 50 μg/mL. The plates were incubated at 37 °C for 12 h, and a single colony was transferred into incubation tubes with 4 mL liquid LB medium and incubated with shaking at 37 °C and 220 rpm until the optical density at 600 nm (OD_600_) reached 0.6–0.8. *Bacillus subtilis* BS168 was transformed with each plasmid via electroporation to obtain the corresponding recombinant strains. Each recombinant strain was inoculated into solid LB plates supplemented with 50 μg/mL kanamycin. The plates were incubated at 37 °C for 12 h, and a single colony was transferred into incubation tubes with 4 mL liquid LB medium and incubated with shaking at 37 °C and 220 rpm until the optical density at 600 nm (OD600) reached 0.6–0.8.

### 2.4. Microbial Surface Expression of Enzymes

Enzyme expression at a small scale was performed as follows: To ensure that the enzymes were expressed on the cell membrane of *Bacillus subtilis*, Western blotting was conducted on both recombinant and wild-type strains using an anti-GGGGS linker rabbit recombinant monoclonal antibody (ProteinTech, Wuhan, China) and an anti-rabbit IgG antibody (Thermo Fisher, Waltham, MA, USA) as the primary and secondary antibodies, respectively. Proteinase K was applied to the recombinant microbes to release sucrose isomerase. A final concentration of 10 μg/mL proteinase K was added to the microbe and incubated at 37 °C for 15 min, followed by the addition of 10 μg AEBSF to end the reaction. The mixture was centrifuged at 12,000 rpm for 10 min, and the supernatant and precipitate were collected separately. Sucrose was then added to each sample at a final concentration of 30 mg/mL. Both the supernatant and precipitate were incubated at 30 °C for 24 h to assess their enzymatic activity. After incubation, the suspensions were centrifuged at 12,000 rpm for 3 min to collect the supernatants. The fluid was diluted 10 times and passed through a 0.22 μm water phase membrane for LC-MS/MS analysis.

For each *Bacillus subtilis* BS168 strain transformed with recombinant plasmids, expression was verified after OD_600_ reached 0.6–0.8 in liquid LB medium, and 10 μL of the suspension was incubated in 3 mL growth medium. For the *Bacillus subtilis* BS168 strain transformed with pMA09S1, glucose and glycerol solutions at an equivalent concentration of 200 mg/mL were added (1 mL) to the medium, with a final concentration of 50 mg/mL, and cultured with shaking at 37 °C and 220 rpm for 24, 48, and 72 h before the enzyme activity assay. For other *Bacillus subtilis* BS168 strains transformed with pMA09S2 to pMA09S29, 200 mg/mL glucose was added (1 mL) to the growth medium after incubation, with a final concentration of 50 mg/mL, and cultured with shaking at 37 °C and 220 rpm before the enzyme activity assay.

### 2.5. Enzyme Activity Assay

Suspensions of growth medium with glucose or glycerol and *Bacillus subtilis* BS168 strains transformed with recombinant plasmids were centrifuged twice at 12,000 rpm for 2 min. After centrifugation, the supernatants were discarded, and the microbial precipitates were washed with 2 mL of polyphosphate buffer solution (PBS), shaken into suspension, and centrifuged again at 12,000 rpm for 2 min. The washing, shaking, and centrifugation steps were repeated thrice. Dry biomass was prepared by concentrating the precipitates using a SpeedVac vacuum concentrator (Thermo Fisher, Waltham, MA, USA). A 5 mL sucrose solution with a concentration of 30 mg/mL was pipetted into 10 mg of dry biomass and shaken to form a suspension. The suspensions were placed for reaction at 30 °C for 24 h and centrifuged at 12,000 rpm for 3 min to collect the reaction fluid as the supernatant. The fluid was diluted 10 times and passed through a 0.22 μm water phase membrane, preparing for LC-MS/MS analysis. All strains were tested in triplicate.

### 2.6. Productivity Analysis

Two *Bacillus subtilis* BS168 strains transformed with pMA09S20 and pMA09S21, respectively, were used in the assay, and their enzyme expression processes were performed as described in Section 2.4 and Section 2.5, respectively. Both suspensions were centrifuged at 12,000 rpm for 2 min, and the precipitates were collected via decantation. Both precipitates were washed and centrifuged three times using the same method before incubation in 5 mL of sucrose at a concentration of 30 mg/mL, shaking into suspension, and placing at 30 °C for the reaction. After 24 h, the suspensions were centrifuged at 12,000 rpm for 3 min to collect the reaction fluids. The remaining precipitate was washed in the same manner before incubation with another 5 mL of sucrose at a concentration of 30 mg/mL [41]. This process was repeated for 7 days, and seven batches of reaction fluids were collected for each strain, diluted 10 times, and passed through a 0.22 μm water phase membrane. A curve of relative enzyme activity versus time was obtained using LC-MS/MS analysis. Both strains were tested in three parallel groups.

### 2.7. Enzymatic Kinetics

*Bacillus subtilis* BS168 strains transformed with pMA09S2, pMA09S20, and pMA09S21 were selected for optimization, and enzyme expression was performed as described in Section 2.4. The suspension was centrifuged at 12,000 rpm for 2 min, and the precipitate was collected. The precipitate was washed and centrifuged thrice using the same method, and 1 g of proteinase K was added to separate the targeted sucrose isomerase from the cell membrane. Enzymatic separation lasted for 15 min at 37 °C, and then 10 μg of AEBSF was added to terminate lysis. The fluid was centrifuged at 12,000 rpm for 2 min, and the supernatants were collected for subsequent analysis. Protein components with molecular weights of 7.5–8.0 kDa were collected by SDS-PAGE separation, and their concentrations were determined using NanoDrop (Thermo Fisher, Waltham, MA, USA). The samples were then concentrated using a SpeedVac vacuum concentrator. The collected components (0.1 mg) were incubated in 5 mL sucrose at different concentrations (0.5, 1, 2, 5, 10, 20, 40, and 60 mg/mL), shaken into suspension, and placed at 30 °C for the reaction. After 10 and 30 min of reaction, 0.1 mL of supernatant was collected from each group after centrifugation at 12,000 rpm for 3 min. All batches of fluids were diluted 10 times and passed through a 0.22 μm water phase membrane for LC-MS/MS analysis. A curve of conversion rate versus sucrose concentration and time was generated. The velocity was determined by the differences in isomaltulose yield between 10 and 30 min and then divided by the 20 min reaction time. Significance levels were compared between each mutant group and the wild type.

### 2.8. Enzyme Thermostability Assessment

Thermostability assessments were conducted for the wild-type enzyme, F181I, and H363P mutants. The procedure was based on previous studies [42]. Briefly, enzyme activity was tested at 30 °C, 40 °C, 50 °C, and 60 °C, at 0, 15, 30, 60, and 120 min after incubating 50 mg sucrose with 0.1 mg enzyme, respectively. The methods for activity determination were the same as those described in Section 2.7. The minimum activity (A_min_) at each temperature was determined when the activity became stable, whereas that at 30 °C was referred to as the native activity (A_0_). A curve of A_min_/A_0_ versus temperature (in Kelvin) was plotted and analyzed using simple linear regression. The melting temperature (T_m_) of each enzyme was determined as the temperature at which the A_min_/A_0_ value was 0.5. The melting temperatures of both mutants were compared with those of the wild-type enzyme.

### 2.9. LC-MS/MS Analysis

The analysis methods for isomaltulose and sucrose were based on previous studies, with some modifications [43]. LC-MS/MS was used to determine isomaltulose with an amide column (Waters Xbridge Amide, 5.0 μm, 4.6 × 250 mm) at 35 °C in a linear gradient elution of acetonitrile and water at 0.8 mL/min: 90:10 (*v*/*v*) from 0 to 6 min, 10:90 (*v*/*v*) from 8 to 14 min, and 90:10 (*v*/*v*) from 16 to 20 min. After HPLC, the samples were delivered to the negative ESI source with a collision voltage of 300 kV and a source temperature of 350 °C. An MRM scan was conducted to identify isomaltulose and sucrose. For isomaltulose, the precursor ion of *m*/*z* 341 and fragment ion pairs of *m*/*z* 101 and 89 were scanned, whereas for sucrose, the precursor ion of *m*/*z* 341 and fragment ion pairs of 221 and 113 were scanned. To quantify the isomaltulose concentration in each sample, standard solutions of 1, 2, 5, 10, 20, and 50 μg/mL were prepared and analyzed under the same conditions to determine the standard curve. Prior to LC-MS/MS analysis, the samples were diluted 10-fold and subjected to 0.22 μm membrane filtration. All tests were performed at least twice.

### 2.10. Statistical Analysis

Data were obtained at least in triplicate and expressed as mean ± standard deviation (SD) (n = 3). Statistical analyses were performed using GraphPad Prism 9.0 with a one-way analysis of variance (ANOVA). Statistical significance was set at *p* < 0.05.

## 3. Results and Discussion

### 3.1. Expression of Wild-Type Sucrose Isomerase

**Figure 2:**
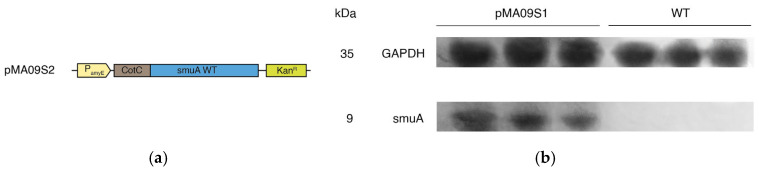

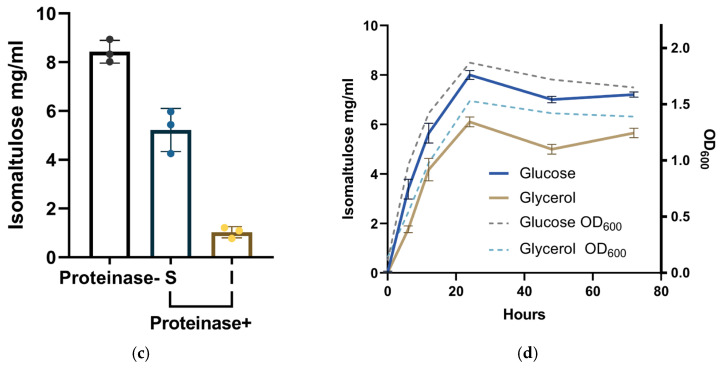
Expression of sucrose isomerase in *Bacillus subtilis*. (**a**) Plasmid map of the originally constructed pMA09S1; (**b**) Western blot of pMA09S1 and wild-type *Bacillus subtilis* strains, using rabbit anti-GGGGS linker antibody; (**c**) isomaltulose yield of pMA09S1 and its components treated with proteinase K for 24 h; (**d**) conversion rate of sucrose into isomaltulose at different cultivation times of *Bacillus subtilis* transformed with pMA09S1 before reaction. S, supernatant; I, insoluble precipitate.

As expected, pMA09S1 was successfully transformed with the *Bacillus subtilis* BS168 wild-type strain, and colonies were observed on kanamycin-supplemented LB plates. In a pre-experiment, sucrose was directly incubated in the growth medium and reacted at 30 °C for 72 h, and approximately 2% of the sucrose was converted into isomaltulose. Western blotting showed the hybridization of the anti-GGGGS antibody with the GGGGS linker, which proved the success of pMA09S1 expression in *Bacillus subtilis* cells (Figure 2a,b). We then applied proteinase K treatment to pMA09S1 strains, with sucrose isomerase activity of 5.53 mg/mL in the supernatant after 24 h, proving its existence on the surface of *Bacillus subtilis* (Figure 2c). To increase the efficacy of sucrose conversion, we explored the optimal cultivation conditions for *Bacillus subtilis* BS168 strains transformed with recombinant plasmids. According to previously reported methods, we selected glucose and glycerol as carbon sources to cultivate transformed microorganisms for 6, 12, 24, 48, and 72 h before enzyme assessment to explore the influence of cultivation conditions on enzyme activity. Compared with glycerol, glucose increased the amount of sucrose isomerase, enhancing reaction efficiency, as more *Bacillus subtilis* grew in the growth medium over the same period (Figure 2d). Interestingly, when comparing enzyme activity at different cultivation times in the same group of carbon sources, we found that the enzyme activity at 24 h of cultivation was the highest, whereas a significant decrease occurred in enzyme activity if the cultivation time was prolonged to 48 h, while it slightly increased as the cultivation time was further prolonged to 72 h. In comparison with OD_600_ at all periods, we assumed that in the first 24 h, *Bacillus subtilis* continued to consume the nutrients in the medium for growth and reproduction. However, at a specific period between 24 and 48 h, as all nutrients in the growth medium were consumed, some microorganisms died of nutrient deficiency, which also reflects the synchronously decreased OD_600_ values at 48 h. However, the OD_600_ values did not decrease continuously at 72 h as the amount of *Bacillus subtilis* reached a steady state, while the expression levels of sucrose isomerase slightly increased over time during this period, but they were not compatible with the levels at 24 h. Thus, we determined that adding 50 mg/mL glucose to the growth medium and 24 h of cultivation was optimal. Under these conditions, the maximum average sucrose conversion rate of *Bacillus subtilis* transformed with pMA09S1 was 27.2%, with an isomaltulose production of 8.99 mg/mL, indicating the need for further optimization.

### 3.2. Optimization of Promoters

**Figure 3:**
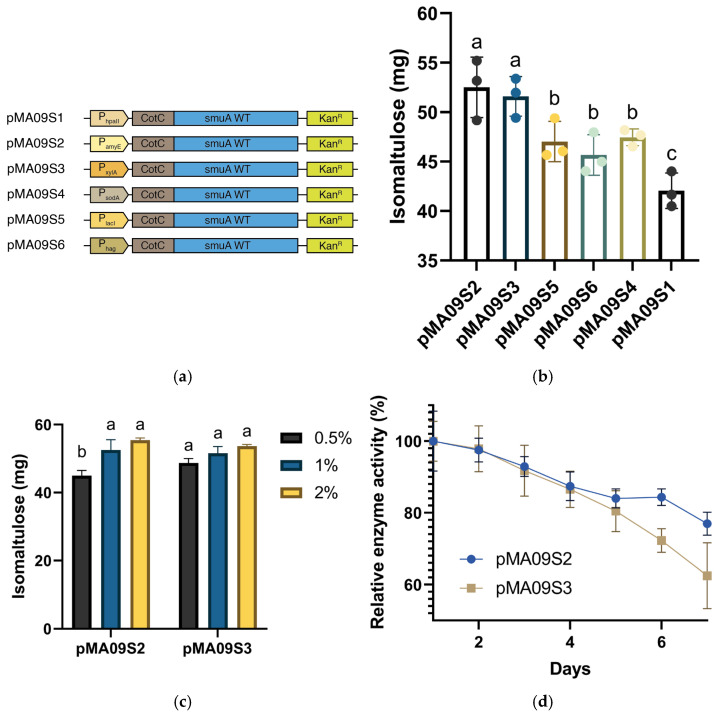
Optimization of promoter in sucrose isomerase-expressing vector. (**a**) Plasmids conducted in promoter optimization; (**b**) net biomass of isomaltulose production from each promoter optimization strain, estimating sucrose isomerase expression levels; (**c**) influence of different concentrations of inducers on sucrose isomerase expression levels of strains transformed with pMA09S2 and pMA09S3 under the same enzyme concentration and cell density; the concentration of inducers is shown as weight/volume (*w*/*v*); (**d**) durability test results of strains transformed with pMA09S2 and pMA09S3. Enzyme activity measured in the first 24 h was taken as 100%. The same concentration of starch and xylose was priorly added as inducer. Significant levels are indicated by lowercase letters above the bars.

We first focused on the promoter of the plasmid, which directly controls the expression of target genes. Because no major influencing factors exist here, the structure of the promoter itself influences the expression of sucrose isomerase. Although the inducer is not necessary, P_hpaII,_ constructed on plasmid pMA09S1, is a weak promoter for *Bacillus subtilis*, and limitations have existed to highly express sucrose isomerase at a large scale. Stronger promoters, such as P_amyE_, P_xylA,_ etc., have shown great ability to enhance the expression levels of targeted genes according to previous reports [34,44,45,46]. We also found P_sodA_, P_lacI_, and P_hag_ to perform well in *Bacillus subtilis* and they were selected as candidate promoters [47,48,49]. We obtained fragments of P_amyE_, P_sodA_, P_xylA_, P_lacI_, and P_hag_ by amplifying their corresponding genomic DNAs and then replaced the original P_hpaII_ promoter, constructing promoter-modified plasmids pMA09S2, pMA09S3, pMA09S4, pMA09S5, and pMA09S6. Five strains, each with a corresponding plasmid, along with the strain containing pMA09S1, were subjected to enzyme activity assays. Since xylose is required to activate P_xylA_, starch for P_amyE_, and IPTG for P_lacI_, 2 mg (1% *w*/*v*) of the corresponding inducer was pipetted at the beginning of cultivation. As shown in Figure 3, all five strains containing promoter-modified plasmids exhibited significantly increased enzymatic activity compared to the strain containing pMA09S1, within which P_hpaII_ was contained when transforming 125 mg of sucrose, showing a maximum yield of 52.63 mg and a conversion rate of 42.1%, achieved by the strain containing pMA09S2. We then compared the promoting levels of the five promoters and found that, compared with P_lacI_ and P_hag_, the rest of the promoters, P_amyE_, P_xylA_, and P_sodA_, showed a greater positive effect on sucrose isomerase expression, while no significance was observed in enzyme activity in the strains with pMA09S2, pMA09S3, and pMA09S4, which contained these three promoters, respectively. Notably, the enzyme activity of the strain with pMA09S4 encoding P_sodA_ as a weak promoter in both *Escherichia coli* and *Bacillus subtilis* was relatively higher than that of the other two weak promoters but showed no significant difference from the strong promoters, namely, P_amyE_ and P_xylA_. It was also discussed previously that P_sodA_ gains its advantage of responding to transcriptional signals even under slight levels of induction [50]. To further determine whether P_amyE_ or P_xylA_ is the optimal promoter for the sucrose isomerase gene, we conducted a durability test for the strains containing pMA09S2 and pMA09S3. Both strains participated in a 7-cycle repeated reaction, during which the former fluids were removed for collection, and 150 mg sucrose in solution was re-incubated every 24 h. The strain containing pMA09S2 maintained a relative enzyme activity of 77% after 7 days when compared to the initial level, whereas the strain containing pMA09S3 could only maintain 64.5% of the initial enzyme activity on day 7 (Figure 3d). The results showed better durability of promoter P_amyE_ than that of P_xylA_, with the former being capable of promoting gene transcription at an elevated level for a longer time, while P_xylA_ showed a relatively shorter range than that of P_amyE_. It is also noticeable that between days 2 and 3, the enzyme activity of the strain containing pMA09S3 surpassed that of the strain containing pMA09S2, whereas after day 4, the enzyme activity of the strain containing pMA09S3 began to decrease rapidly. This result also matches previous research, showing that P_xylA_ could strongly enhance gene transcription in a short time; however, P_amyE_ may continuously enhance transcription levels with a larger integrated transcription level [51,52]. To investigate whether a higher concentration of inducers may increase enzyme production, we added 0.5%, 1%, and 2% (*w*/*v*) of starch and xylose, respectively, into the culture medium of strains containing pMA09S2 and pMA09S3. The results shown in Figure 3c indicate that continuously increasing the concentration of inducers does not significantly increase the promotion levels of either promoter, while with a higher xylose concentration, the promotion level of P_xylA_ shall certainly not surpass that of P_amyE_.

### 3.3. Optimization of Spore Coat Protein

**Figure 4:**
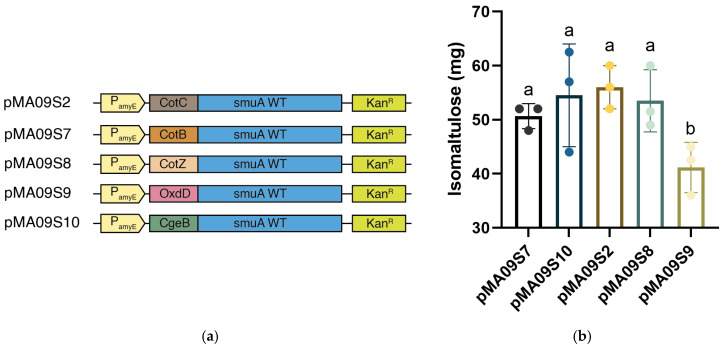
Optimization of spore coat proteins in sucrose isomerase-expressing vectors. (**a**) Plasmids constructed for optimization of spore coat proteins; (**b**) net biomass of isomaltulose production from spore coat protein optimization strains, estimating sucrose isomerase expression levels. Significant levels are indicated by lowercase letters above the bars.

Having determined P_amyE_ as the optimal promoter for sucrose isomerase, we focused on the selection of spore coat proteins for surface display. In *Bacillus subtilis*, spore coat proteins with higher expression levels contribute significantly to the microbial surface display capacity of target proteins, indicating an increased chance of the target protein being displayed on the cell membrane [23]. In addition to the originally applied CotC, a series of alternatives consisting of CotB, CotZ, OxdD, and CgeA were investigated and determined to be candidate promoters. The transmembrane motif nucleotides of the alternative spore coat proteins were obtained by amplifying *Bacillus subtilis* genomic DNA with designed primers, and the *cotC* fragment of pMA09S2 was replaced with the respective nucleotides encoding CotB, CotZ, OxdD, and CgeA, resulting in pMA09S7 to pMA09S10 (Figure 4a). We conducted enzyme activity assays for plasmids encoding pMA09S7 to pMA09S10, along with pMA09S2, using the same method, and the results are displayed in Figure 4b. We found that the primarily applied spore coat protein CotC displayed the best immobilization level for sucrose isomerase among all candidates. This is not only because CotC is more abundant in *Bacillus subtilis* spore coats than CotB [21], but also because previous experiments have shown that CotC shows great display efficiency for target proteins via specific amino terminal fusions, by enhancing transcriptional and translational signals of recombinant vectors [53]. Another study has shown that the translation of spore coat proteins in *Bacillus subtilis* is mediated by cultivation temperature, which responds to the activity of the kinase CotH. CotH is a *Bacillus subtilis* spore coat protein kinase that catalyzes the formation of the phosphorylated forms of CotZ and CotB for aggravation, and its activity decreases as the temperature increases [54]. At a lower temperature of approximately 25 °C, CotZ mostly exhibits its phosphorylated form, which is aggravated. Under such circumstances, *cotZ* and *cotB* gain the majority of transcription and translation levels, whereas *cotC* expression levels are inhibited. As the cultivation temperature increases above 37 °C, the activity of CotH fades, and a large fraction of CotZ and CotB remains in their unphosphorylated and unaggravated forms, with decreased expression levels of *cotZ* and *cotB* but a higher level of *cotC*. Consequently, all spore coat proteins share a balanced expression level under 37 °C cultivation, while *cotC* shares the majority of the expression. Mature CotZ and CotB completely disappear at 42 °C; however, the level of CotC does not continuously increase as the temperature rises, which is not practical for our current study [55,56]. It can be concluded that high initial efficiency and an appropriate cultivation temperature both contribute to the outstanding display efficiency of CotC for sucrose isomerase.

### 3.4. Mutation Selection of Sucrose Isomerase and Kinetics Assay

**Figure 5:**
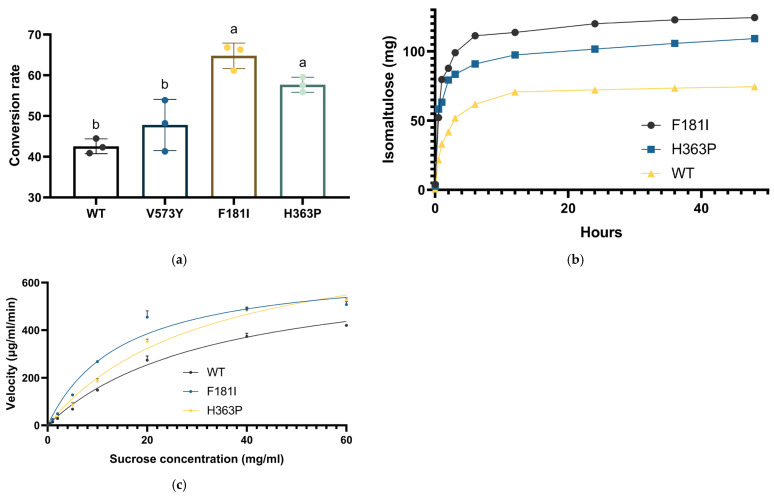
Conversion rate of sucrose to isomaltulose in strains with plasmids encoding mutant enzyme genes and plasmids encoding wild-type enzyme genes, indicating levels of sucrose isomerase activity. (**a**) Assessment of pMA09S11, pMA09S20, and pMA09S21, expressing wild-type, V573Y-mutant, F181I-mutant, and H363P-mutant enzymes, respectively, at levels above those of pMA09S2 during the pre-assessment; (**b**) productivity plot of isomaltulose production versus reaction time; (**c**) dynamic curve of wild-type, F181I-mutant, and H363P-mutant sucrose isomerase showing initial catalyst velocity versus sucrose concentration. WT—wild type. Significant levels are indicated by lowercase letters above the bars.

An enzyme structure that adopts the substrate is considered to gain better catalyst efficiency, reflected by the enzymatic capacity and reaction rate, which are reflected by two constants, k_cat_ and K_m_. In terms of enzyme kinetics, a larger k_cat_ indicates a higher ratio of substrate conversion, whereas a lower K_m_ indicates a higher affinity of the substrate for the enzyme. Here, a higher sucrose conversion rate is required, and modification of sucrose isomerase towards a higher k_cat_ value is vital. We introduced site-specific mutations into the wild-type sucrose isomerase to modify its active center and ensure that its catalyst motif exhibited better binding with sucrose [57]. Sucrose isomerase is a transmembrane protein located in the inner membrane of *Serratia plymuthica*. Its backbone consists of two motifs: a transmembrane motif with loosely hydrophobic α-folds and a hydrophilic catalyst motif, the latter of which is the catalyst site. A high binding level with the substrate indicates higher catalyst efficiency, and the interactions binding with the substrate mainly consist of ionic forces, hydrophobic bonds, van der Waals forces, and hydrogen bonds [16,58]. We constructed a molecular model of sucrose isomerase to observe its catalytic center and explore the arrangement of residues. According to the docking results of wild-type sucrose isomerase and sucrose, we determined 19 single-point mutant candidates, whose binding energy with sucrose was below zero: V573Y, M129V, H94F, L192Y, H363P, F43Q, R150P, Y168W, Y19A, F181I, F405Y, F431A, R344K, F479L, M83A, N130K, A292V, R148Q, and N465D (Table S3). The corresponding plasmids encoding the mutants were constructed by amplifying single-point mutation pairs, obtaining pMA09S11 to pMA09S29. All mutants were expressed in cells, and the strains underwent pre-selection using identical assay methods as previously described (Figure S1). Among the 19 mutants, V573Y, F181I, and H363P, which corresponded to strains pMA09S11, pMA09S20, and pMA09S21, respectively, exhibited a higher sucrose conversion rate than that of the wild type. We next investigated three selected potential candidates, V573Y, F181I, and F405Y, each conducted in triplicate to exclude random interference, and found that strains expressing F168I and H363P showed a significant increase in sucrose conversion rate, reaching 66.65% and 56.47%, respectively. The production of isomaltulose of two strains attained 19.98 mg/mL and 16.94 mg/mL, of which strain pMA09S20 was higher in significance, while pMA09S11 expressing the V573Y mutant did not show a significant increase in sucrose conversion rate, acquiring an ultimate conversion rate of 47.80% (Figure 5a). In particular, the productivity of isomaltulose rapidly increased in both mutants within 12 h of incubation. The yield of isomaltulose in the F181I mutant reached >100 mg within 3 h, while the yield in the H363P mutant also reached 90 mg after 6 h. The maximum yield also exhibited a noticeable increase in both mutants, being 123.2 mg in F181I and 104.1 mg in H363P, while the yield in the wild-type enzyme was 61.7 mg (Figure 5b). To investigate the mechanisms of the two mutants, we conducted dynamic assays for both mutants along with the wild type. A velocity versus substrate concentration curve was plotted to determine the values of k_cat_ and K_m_. The results showed that the F181I-mutant did not significantly increase catalyst capacity, as indicated by the value of k_cat_, which was 17.64 s^−1^ for wild type and 17.46 s^−1^ for F181I-mutant, and did not exhibit a significant difference at that specific point, although K_m_ decreased noticeably from 33.38 mg/mL to 14.97 mg/mL. A different mechanism was exhibited in the H363P mutant, which showed a slightly but significantly increased k_cat_ of 24.80 s^−1^ (Figure 5c and Table 1).

**Figure 6:**
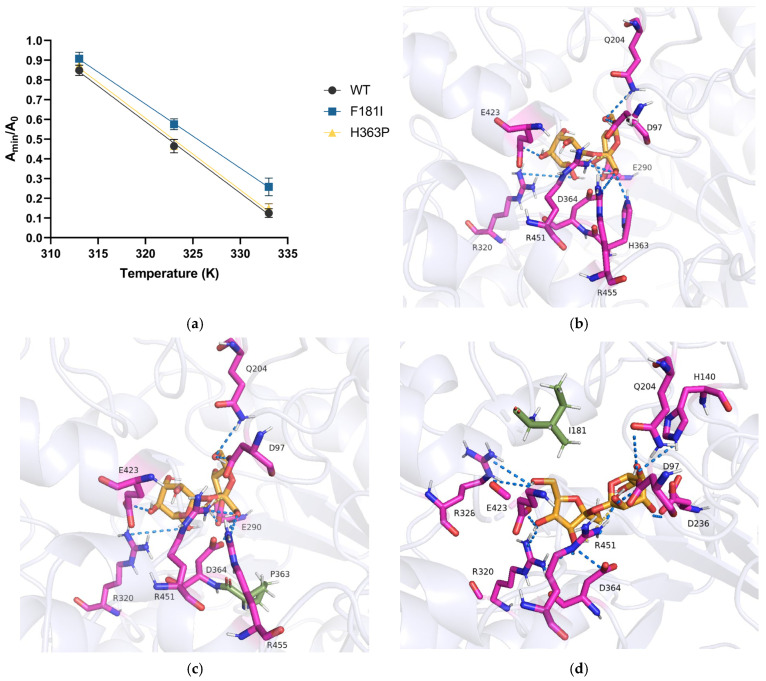
Thermostability and docking results of the wild-type, H363P, and F181I mutants. (**a**) A_min_/A_0_ versus temperature curve, where the melting temperature is defined as the temperature at which the enzyme maintains half of the activity of the native enzyme (A_min_/A_0_ = 0.5); (**b**) docking results of sucrose and wild-type enzyme; (**c**) docking results of sucrose and H363P mutant, where the residue in green represents the mutation site; and (**d**) docking results of sucrose and F181I mutant, where the residue in green represents the mutation site. Molecular interactions are shown as dotted lines, with blue for hydrogen bonds, and yellow for salt bridges.

The increase in volumetric productivity can be normally contributed to the rise in k_cat_, the increase in enzyme stability, or the reduction in product inhibition [41]. H363P mutant has shown an increase in experimental k_cat;_ however, k_cat_ of F181I mutant had no significance compared to the wild type. To determine whether the stability of the F181I mutant increased, we conducted a thermostability assay for the enzymes as previously described. Melting temperature (T_m_) stands for the temperature where the minimum activity level (A_min_) of the enzyme is half of that at its optimal temperature, whereby the value of melting temperature reflects the thermostability of enzyme. We tested the minimum activity of the wild-type and two mutants at 40 °C, 50 °C, and 60 °C and compared them with the minimum activity at 30 °C. The wild-type enzyme performed a lower thermostability: its activity decreased rapidly as the temperature rose, having only 13.4% of its initial activity remaining at 60 °C as compared to the activity at 30 °C, along with a melting temperature estimated to be 49.5 °C, which matches previous studies [59,60]. H363P has also shown a similar thermostability level as the wild type, with a minimum activity of 14.7% at 60 °C, and a melting temperature of 49.8 °C. F181I mutant, on the contrary, has exhibited an improved stability. Around 25.4% of the initial activity has been preserved at 60 °C, while its melting temperature was 52.5 °C, making a remarkable increase from wild type (Figure 6a). It can also be interpreted from the structure of the sucrose isomerase, as the histidine at the 363rd position takes part in the formation of its catalyst center [61]. Sucrose binds with the wild-type enzyme mainly via hydrogen bonds and salt bridges connecting with residues at the catalyst center, namely R97, A290, R320, D364, E423, and R451, contributing to an acceptable binding energy of −5.13 kcal/mol (Figure 6b), while hydrogen bonds have proved to be a major factor in stabilizing the enzyme and increasing affinity to sucrose [58]. Mutation of the H363 site does not significantly influence the intermolecular electrostatic interaction of the enzyme with sucrose, as the binding residues have remained the same as in the wild type, with a hydrogen bond primarily linked to H363 having shifted to D364. The binding energy of H363P was −5.15 kcal/mol, which has no significance to that of WT. This is also reflected by its thermostability that has remained similar to that of the wild type, as well as K_m_ (Figure 6c). The modification to the histidine residue has contributed to an increased k_cat_, possibly due to a change in the size and shape of the catalyst center, while mutations of basic amino acid residues (H or R) may also contribute to the shift in enzyme kinetics [59]. Still, the H363P mutation only increases k_cat_ but does not enhance the affinity to sucrose and the stability of the enzyme; it is likely to become “saturated”, especially at a higher sucrose concentration, resulting in a relatively longer period for the productivity to reach its plateau. In contrast, the F181I mutation has enabled sucrose to exhibit a more stable conformation when binding to enzymes. The binding site in the catalyst center has slightly shifted but has provided access to more electrostatic interactions. 15 hydrogen bonds and a salt bridge in total have formed between F181I mutant and sucrose, lowering the binding energy to −6.23 kcal/mol, stabilizing the enzyme–substrate complex (Figure 6d). Since the K_m_ in the F181I mutant has decreased compared to that in the wild-type enzyme, it is capable of reaching a higher efficiency in sucrose binding and utilization, resulting in an elevated velocity and productivity, while a shortened period is required to reach its plateau.

Although the F181I-mutant can reach a stable maximum velocity at a lower sucrose concentration because of its stabilized conformation, which indicates that further increasing the conversion rate by lowering the sucrose concentration seems possible, the conversion rate does not significantly increase, which is 69.6% at 30 mg/mL and 72.2% at 20 mg/mL. In contrast, because the catalyst velocity had not yet reached the limit, increasing the sucrose concentration rather than extending the reaction time was beneficial for improving isomaltulose production, albeit with a stable conversion rate and a slight decrease.

The yield of isomaltulose increased to 27.21 mg/mL as the sucrose concentration increased to 40 mg/mL, although the conversion rate decreased slightly to 68.0%. However, the catalyst velocity reached a maximum when the sucrose concentration increased above 40%, resulting in stable production and a decreased conversion rate (Table 2). This occurs not only in the F181I mutant but also in the H363P mutant and the wild type. These results suggest that increasing the sucrose concentration to 40 mg/mL is beneficial for large-scale production, as a remarkable substrate inhibition effect has occurred at high sucrose concentrations. Substrate inhibition is commonly observed in sucrose isomerases, covering various wild-type enzymes to recombinant enzymes, presumably due to the high viscosity of the sucrose solution, leading to a decrease in mass-transfer efficiency and enzyme kinetics; subsequently, product inhibition is also common in sucrose isomerases, sharing a similar mechanism [16,62,63]. In conclusion, the F181I and H363P mutants improve the catalyst efficiency of sucrose isomerase from different perspectives. By removing large, bulky, barrier-functioning residues at the catalyst pocket, an attenuated binding condition was achieved in the F181I-mutant with no significant improvement in conversion velocity. Modifying the catalyst motif in the H363P-mutant improved conversion but did not significantly affect sucrose binding.

Through rounds of vector optimization and mutant design, we determined the optimal surface-displaying system of sucrose isomerase in *Bacillus subtilis*. The productivity and conversion rate have improved considerably when compared with those of the wild type and have shown a good level among other surface-displaying systems in *Bacillus subtilis*. The two designed mutants, F181I and H363P, are considered helpful to provide reasoning to design mutants of sucrose isomerases from alternative origins. Moreover, the procedure for constructing the surface-displaying system and the cultivation of *Bacillus subtilis* are relatively simple, making it convenient for large-scale production. However, improvements are also necessary to meet the productivity standards of yeast cells. We predict that the construction of double-site mutations at both points will contribute to improved catalyst properties with an increased k_cat_/K_m_ ratio, which will be investigated in future studies.

## 4. Conclusions

In the current study, sucrose isomerase *smuA* from *Serratia plymuthica* was introduced and expressed in *Bacillus subtilis* BS168. Optimization of the carbon source, promoter, and spore coat protein significantly improved the yield of isomerase to 14.52 mg/mL and the conversion rate to 48.4%. Site-specific mutation design of sucrose isomerase at the F181 site increased the isomaltulose yield to a maximum value of 27.21 mg/mL and a maximum conversion rate of 72.2% by enhancing the stability of the enzyme–substrate complex. The optimally optimized strains expressing F181I- and H363P-mutant sucrose isomerase are capable of producing isomaltulose on a large scale for the food industry with a wider range of sucrose initial concentrations, which is an outstanding advantage in microbial-based isomaltulose cell factories. In the future, we will focus on further optimizations, such as promoter and multi-spot mutations of sucrose isomerase, to improve the isomaltulose yield.

## Figures and Tables

**Table 1:** Catalyst constants of sucrose isomerase expressed by pMA09S2, pMA09S20, and pMA09S21 (mean ± SD, n = 3).

Constant	WT	F181I	H363P
V_max_ (μg/mL/min)	679.1 ± 37.3 ^b^	672.2 ± 26.5 ^b^	839.2 ± 30.5 ^a^
k_cat_ (1/s)	17.64 ± 2.08 ^b^	17.46 ± 1.94 ^b^	24.80 ± 2.37 ^a^
K_m_ (μg/mL)	33.38 ± 2.40 ^a^	14.97 ± 0.92 ^b^	31.98 ± 1.43 ^a^
k_cat_/K_m_	5.285 ± 2.41 ^a^	11.71 ± 3.84 ^b^	7.754 ± 4.28 ^ab^

Note: Significant levels are indicated by lowercase letters.

**Table 2:** Catalyst constants of sucrose isomerase expressed by pMA09S20 at different sucrose concentrations (mean ± SD, n = 3).

Sucrose Concentration (mg/mL)	Isomaltulose Concentration (mg/mL)	Conversion Rate (%)
20	14.44 ± 3.37	72.2 ^a^
25	17.65 ± 2.90	70.6 ^a^
30	20.88 ± 5.06	69.6 ^a^
35	24.10 ± 4.89	68.9 ^a^
40	27.21 ± 6.02	68.0 ^a^
50	28.06 ± 6.31	56.1 ^b^

Note: Significant levels are indicated by lowercase letters.

## Data Availability

Due to privacy, the data presented in this study are available on request from the corresponding author.

## Supplementary Materials

The following supporting information can be downloaded at: https://www.mdpi.com/article/10.3390/microorganisms14040817/s1, Figure S1, Conversion rate of wild type and mutant sucrose isomerases at an enzyme–substrate ratio (*w*/*w*) of 1/100. All reactions were incubated for 24 h at 30 °C and 200 rpm. WT—wild type; Figure S2, Conversion rate of sucrose of wild-type, F181I-, and H363P-mutated sucrose isomerases at an enzyme–substrate ratio (*w*/*w*) of 1:100. All reactions were incubated for 24 h at 30 °C and 200 rpm. WT—wild type; Figure S3: Western blot gel of pMA09S1 (Lanes 1 to 3) and wild-type Bacillus subtilis (Lanes 4 to 6); Table S1, Amplification target mutants of each primer pair; Table S2: Sequences of primers used in this study; Table S3: Computed binding energies of wild-type and mutants with sucrose.
