# Supplementary material for: Sucrose Isomerase Mutants’ Expression in Bacillus subtilis for Isomaltulose Production
Source: Microorganisms. 2026 Apr 2;14(4):817. doi: 10.3390/microorganisms14040817 (PMC13119103; doi:10.3390/microorganisms14040817)
Supplement: Supplementary file 1 [file microorganisms-14-00817-s001.zip › microorganisms-4168459-supplementary.pdf]

Sucrose Isomerase Mutants Expression in *Bacillus subtilis* for Isomaltulose Production

Supplementary Materials

Table S1 Amplification target mutants of each primer pair

Primer pair	Vector	*smuA* mutant
PM01/02	pMA09S11	V573Y
PM03/04	pMA09S12	M129V
PM05/06	pMA09S13	H94F
PM07/08	pMA09S14	L192Y
PM09/10	pMA09S15	F405Y
PM11/12	pMA09S16	F43Q
PM13/14	pMA09S17	R150P
PM15/16	pMA09S18	Y168W
PM17/18	pMA09S19	Y19A
PM19/20	pMA09S20	F181I
PM21/22	pMA09S21	H363P
PM23/24	pMA09S22	F431A
PM25/26	pMA09S23	R344K
PM27/28	pMA09S24	F479L
PM29/30	pMA09S25	M83A
PM31/32	pMA09S26	N130K
PM33/34	pMA09S27	A292V
PM35/36	pMA09S28	R148Q
PM37/38	pMA09S29	N465D

Table S2 Sequences of primers used in this study

Name	Sequence (5'→3')
P01	TTAATGGGTTATTACAAAAAATACAAAGAAGAG
P02	AAAAATCTCCACCTTTAAACCC
P03	GGAGTGTCAAGAATGGGTTATTACAAAA
P04	TCTTGACACTCCTTATTTGATTT
P05	GGAGGAATTATTCATGGGTTATTACAAAAAAT
P06	CTTATAACTCAAAAAATCTCCACCTTTAAACC
P07	GAAATGGGATCCTCTATGGGTTATTACAAAAA
P08	TGTTCTCAAAAAATCTCCACCTT
P09	CGCCCGGATGGGTTATTA
P10	CAACGCTCAAAAAATCTCCACC
P11	GAGGAACAAAACAATGGGTTATTA
P12	TTATCGATCTCAAAAAATCTCCACC
P13	GGCGGCGGCTCAATGTTTTTGAATGGCTTTAAA
P14	TCTTGACACTCCTTATTTGATTT
P15	TAAATTTGGTGGCGGC
P16	TCTCCTCTTGCTCATTCTTGACAC
P17	ATACAAAGGTGGCGGCGG
P18	TGGGAATAGTGGCCCAATCTTG
P19	AATATCCCGGTACGGGTG
P20	TGTTCCAACAGCATTCTTGACA
P21	TCTTTTCAGGTGGCGGCG
P22	TTCAGAGCTCATTCTTGACACTCC
PM1	TTATCATCAGCCTTCTCTGCAAGAAAACGCGT
PM2	GAGAAGGCTGATGATAATTTTCAAGTAAACTAGATGCAATGGTA
PM3	GTCTAGTTGCTGAGTTGAATAAAAGAGGCATGAGACTAATGATTG
PM4	CAACTCAGCAACTAGACGGTCGAAATCAGCCA
PM5	CCGTTCTATGAATCTCCAAACACGGATAATGG
PM6	GGAGATTCATAGAACGGATTAATCCAGATTGCATCA
PM7	TTGGCAATACGACAAGCAAACAGATCAATATTATTTGC
PM8	GCTTGTCGTATTGCCAAGCGGACCCACCGAAA
PM9	TCAGCCGCTTTGGCGATGACTCACCTCAATGG
PM10	ATCGCCAAAGCGGCTGACCTGCCTCGGGTTAT
PM11	AGCAGACCAACCGATTTGGTGGAAACAAGCCG
PM12	AAATCGGTTGGTCTGCTGATTTTTGAATATTTGTCG
PM13	AGGCACCCAAGATGGTTCGTTCAAAGCCGCTC
PM14	AACCATCTTGGGTGCCTGTCAGATGTATGATTAATGA
PM15	GAACAACTGGCCTTCGTTTTTCGGTGGGTCCG
PM16	ACGAAGGCCAGTTGTTCGGCGCCTGTCCCTGC
PM17	TCCTCTTTCGCTCTCGCCGCTAGCCCGCTTAC
PM18	GCGAGAGCGAAAGAGGACGCCATAGTCAGTGC

PM19	ATATTTGGAGAGACGGCAAGCAGGGACAGGCG
PM20	GCCGTCTCTCCAAATATAGTAGTCACGATAGGGATTGTCCTT
PM21	CCGTTTATTTATCAAGGAGCAGAATTGGGAATGA
PM22	CCTTGATAAATAAACGGTGTTGCTCTTTGTGT
PM23	GAAAGGTGCTTGGAATGATTATGTAGCCTCAGGC
PM24	CATTCCAAGCACCTTTCACTTCAATATCATCGAACT
PM25	GGTAAGCAAGTCATCTCCCAGACGGATCGTGC
PM26	GAGATGACTTGCTTACCTTTCACTTCAATATCATCGAACT
PM27	GCCATGGCTCTTCCATTTAAATCCCAACTACAAACA
PM28	AATGGAAGAGCCATGGCTTCCCTTGCGTAAAA
PM29	CTACTTAAAAGCGTTCCATTTAAATCCCAACTACAAACA
PM30	GGAACGCTTTTAAGTAGTCTAACTTCTCGATAATGCC
PM31	GAGATGAAAAAAAGAGGCATGAGACTAATGATTGA
PM32	CCTCTTTTTTTCATCTCAGCAACTAGACGGTCG
PM33	ACCGTCGGCGAAATCTTCGGGGTGCCGGTAAG
PM34	AAGATTTCGCCGACGGTGGCCACATTATATTTAGAAAGC
PM35	ATCTGACCAGCACAGATGGTTCGTTCAAAGCC
PM36	ATCTGTGCTGGTCAGATGTATGATTAATGACGATATCAA
PM37	ATGCAGTGGGACGACTCCGTCAATGCGGGTTT
PM38	GAGTCGTCCCACTGCATCGGCGTCCGCGAATT

Table S3. Computed binding energies of wild-type and mutants with sucrose.

Enzyme	Binding energy of most populated cluster(kcal/mol)
WT	-5.13
V573Y	-4.86
M129V	-5.69
H94F	-5.44
L192Y	-4.70
H363P	-5.15
F43Q	-5.01
R150P	-5.14
Y168W	-4.33
Y19A	-5.11
F181I	-6.23
F405Y	-5.56
F431A	-5.40
R344K	-5.35
F479L	-5.23
M83A	-4.75
N130K	-4.98
A292V	-4.55
R148Q	-5.10
N465D	-5.45

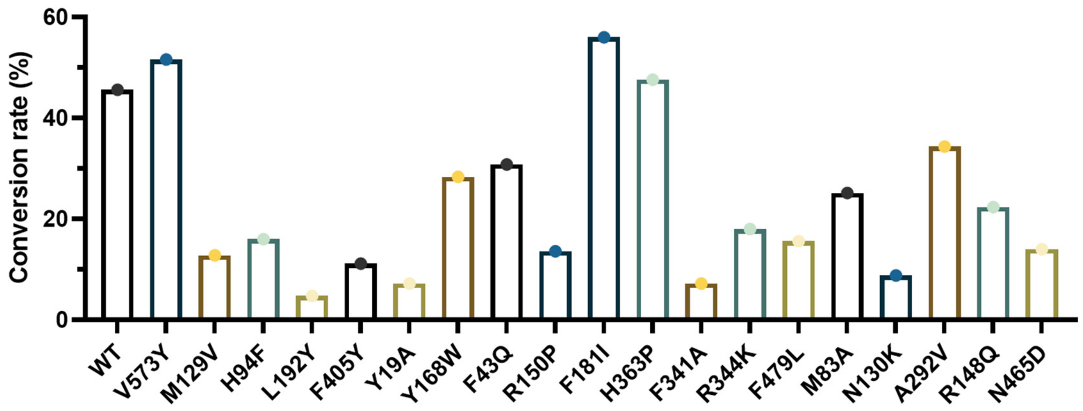

Figure S1. Conversion rate of wild type and mutant sucrose isomerases at an enzyme-substrate ratio (w/w) of 1/100. All reactions were lasted for 24 hours at 30°C and 200 rpm. WT – wild type.

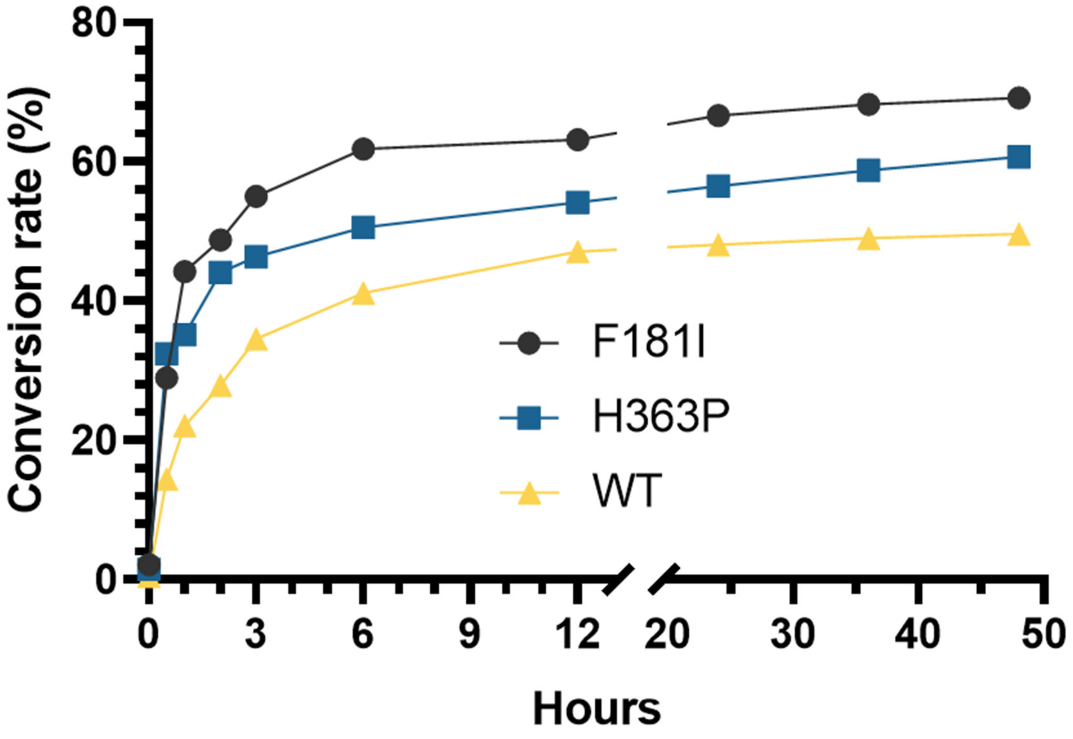

Figure S2. Conversion rate of sucrose of wild type, F181I- and H363P-mutated sucrose isomerases at an enzyme-substrate ratio (w/w) of 1/100. All reactions were lasted for 24 hours at 30°C and 200 rpm. WT – wild type.

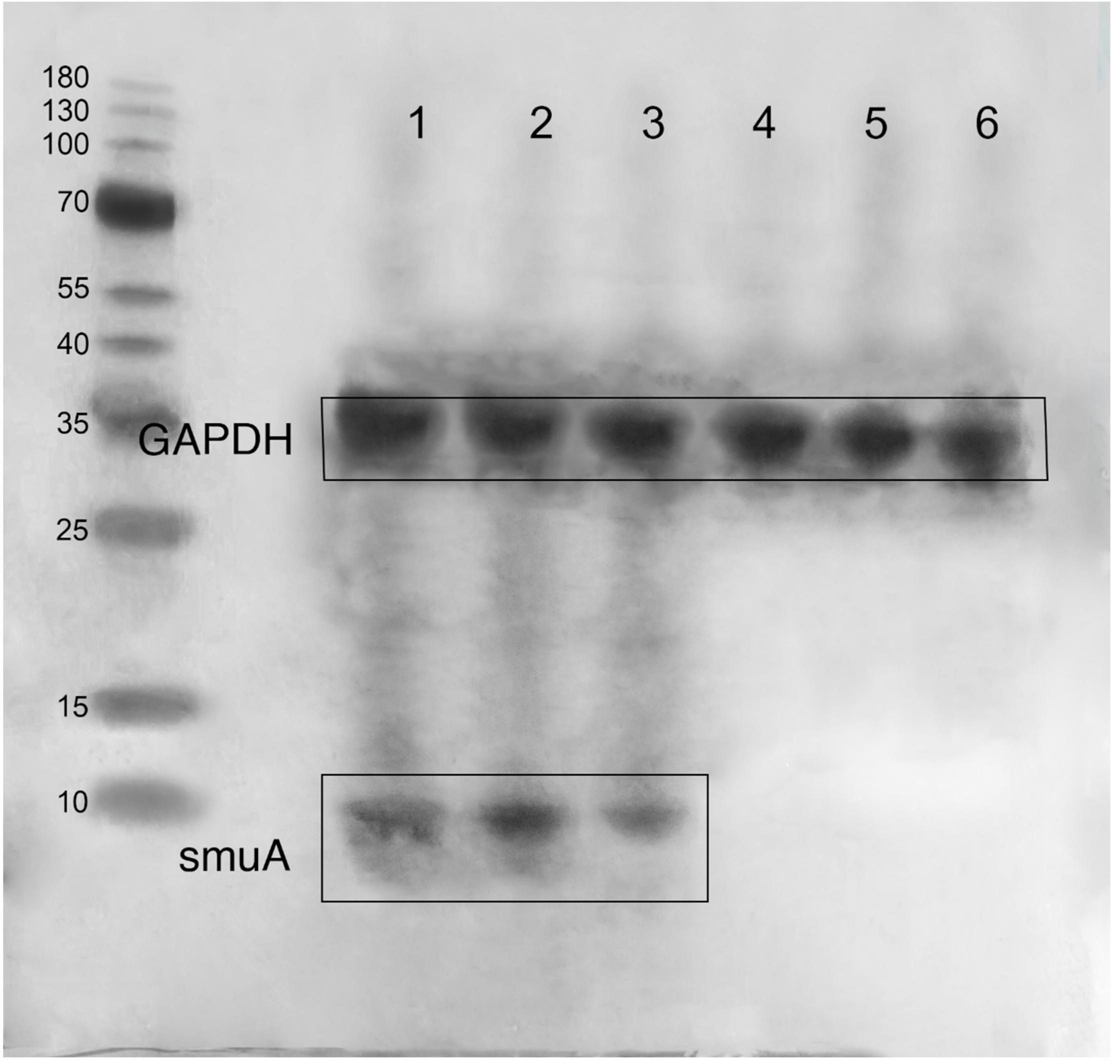

Figure S3. Western blot gel of pMA09S1 (Lanes 1 to 3) and wild-type Bacillus subtilis (Lanes 4 to 6).
